# Circ_0081343 promotes autophagy and alleviates pyroptosis via PI3 K/AKT/HIF-1α axis in hypoxia-induced fetal growth restriction of mice

**DOI:** 10.1080/19768354.2025.2498932

**Published:** 2025-05-06

**Authors:** Linmei Zheng, Rong Tang, Fiaz Ahmad, Junbo Fang, Lei Shi, Xiaoju Chen, Jing Li

**Affiliations:** a Department of Obstetrics, Hainan General Hospital, Hainan Affiliated Hospital of Hainan Medical University, Haikou, People’s Republic of China; b Department of General Surgery, Hainan General Hospital, Hainan Affiliated Hospital of Hainan Medical University, Haikou, People’s Republic of China; c Key Laboratory for Space Biosciences and Biotechnology, School of Life Sciences, Northwestern Polytechnical University (NPU), Xi'an, People’s Republic of China; d Department of Pathology, Southern Medical University, Guangzhou, People’s Republic of China; e Department of Obstetrics and Gynecology, Nanfang Hospital, Southern Medical University, Guangzhou, People’s Republic of China

**Keywords:** Fetal growth restriction, Circ_0081343, NLRP3, Pyroptosis, Placenta

## Abstract

Objective: Fetal growth restriction (FGR) is a serious pregnancy complication associated with an increased risk of perinatal morbidity and mortality. Notably, circular RNAs (circRNAs) significantly influence physiological development and disease pathogenesis. We reported previously that lower circ_0081343 expression is associated with placental trophoblast dysfunction. However, only a few studies have reported the role of circRNAs in FGR in vivo. Therefore, we investigated the effects of circ_0081343 overexpression in the FGR mouse model induced by maternal hypoxia. Methods: Pregnant C57BL/6 mice were kept under hypoxic conditions (10.5% O2) from gestational days 11–17.5, whereas control mice were kept in normal oxygen conditions throughout the gestation period. The animals were sacrificed on the 18.5th day of gestation for prenatal observation. We recorded the maternal body weight, fetal body weight, crown-rump length, and placental weight. Subsequently, we assessed the expression of autophagy, pyroptosis-related protein, and PI3 K/AKT/HIF-1α pathway molecules in placental tissues using RT-PCR, western blotting, ELISA, and immunohistochemistry analysis. Results: We observed low mmu_circ_0081343 expression in the placental tissues of the FGR mouse. However, the expression increased following the injection of adenovirus-mmu-circ_0081343. The overexpression of mmu-circ_0081343 alleviated FGR symptoms in the pregnant mice, including increasing fetal body and placental weight and ameliorating histological injury of the placenta. Additionally, overexpression of mmu-circ_0081343 upregulated Beclin1 expression, increased the LC3II/I ratio, and downregulated P62 expression, while suppressing the PI3 K/AKT/HIF-1α pathway. Conclusions: circ_0081343 alleviated gestational hypoxia-induced placental dysfunction and fetal growth restriction (FGR) by promoting autophagy and inhibiting pyroptosis, potentially through the PI3 K/AKT/HIF-1α pathway.

## Introduction

Fetal growth restriction (FGR), defined as the failure of a fetus to reach its genetically determined potential size, is associated with perinatal morbidity, mortality, and lifelong health risks (Pels et al. 2020). Accumulating evidence indicates that approximately 40% of FGR cases are idiopathic, with their underlying causes remaining unknown (Wu et al. 2017). For these idiopathic FGR cases, effective screening, prevention, and management strategies are still lacking. Previous research has revealed that hypoxia-induced uteroplacental insufficiency or placental dysfunction is now recognized as a key pathological factor in FGR (Nardozza et al. 2017). However, the molecular mechanisms of placental dysfunction-induced FGR remain unclear.

Hypoxia may lead to the alteration of transcripts, including coding RNAs and non-coding RNAs. A newly identified class of noncoding RNAs known as circular RNAs (circRNAs) has a closed loop structure, lacking 5′ caps and 3′ ends that is crucial in the complex gene regulatory network for embryogenesis and cell differentiation (Ren et al. 2023). Emerging studies have demonstrated that circRNAs play critical roles in diverse pathobiological processes, functioning as potential disease biomarkers, acting as competing endogenous RNAs (ceRNAs), interacting with RNA-binding proteins (RBPs), and being translated into functional peptides (Zhou et al. 2020). An increasing number of studies have demonstrated that circRNAs are involved in the occurrence and development of hypoxia-induced FGR. Zhou et al. utilized RNA-seq to assess the expression profiles of circRNAs in human chorionic trophoblast cells under both normal oxygen and hypoxic conditions, identifying a total of 41 differentially expressed circRNAs (Zhou et al. 2024). Yang et al. (2024) found that the expression level of CircCUL1 was significantly increased in FGR placentas and contributed to the development of FGR by suppressing trophoblast cell migration and invasion while promoting autophagy. Additionally, the expression of circTHBS1 was downregulated in FGR placentas, where it functioned as a molecular sponge for miR-136-3p (Guo et al. 2024).

Autophagy and pyroptosis are two interconnected cellular processes that play pivotal roles in maintaining cellular homeostasis and responding to stress (Zhao et al. 2022). Autophagy is a critical cellular response to hypoxia, acting as a survival mechanism to mitigate the adverse effects of oxygen deprivation. Under hypoxic conditions, cells activate autophagy to degrade and recycle damaged organelles, misfolded proteins, and other cellular debris, thereby maintaining energy homeostasis and reducing oxidative stress (Wu et al. 2015). In contrast, pyroptosis is an inflammatory form of programmed cell death triggered by severe or prolonged hypoxia. It involves the activation of inflammasomes, leading to cell membrane rupture and the release of pro-inflammatory cytokines, which can exacerbate cell damage (Meybodi et al. 2024). Interestingly, these processes can influence each other; for instance, autophagy can suppress pyroptosis by degrading inflammasome components or damaged mitochondria that might otherwise trigger pyroptotic pathways. Conversely, excessive or dysregulated autophagy can lead to cellular stress, potentially activating pyroptosis (Zhao et al. 2022). However, it remains unclear whether circRNAs regulate autophagy and pyroptosis in the context of FGR, a condition often associated with chronic hypoxia during pregnancy.

Here, animal experiments were conducted to evaluate the role of circ_0081343 in FGR. In the current study, we investigated the hypothesis that abnormal expression of circ_0081343 in mouse fetuses could negatively affect fetal development under conditions of chronic hypoxia during pregnancy. Our aim was to determine the protective role of circ_0081343 against hypoxia-induced impairment in fetal development in mice with FGR, and to identify a potential predictive and therapeutic target for FGR treatment.

## Methods

### Quantitative real-time quantitative reverse transcription polymerase chain reaction

We extracted the total RNA from murine placentas using Trizol (Invitrogen, Waltham, MA, USA) following the manufacturer’s instructions. Subsequently, a NanoDrop spectrophotometer (Thermo Fisher Scientific, USA) was used to quantify the total RNAs. Reverse transcription of 1μg of total RNA into cDNA was performed using PrimeScript RT Master Mix (TaKaRa, Dalian, China). quantitative reverse transcription polymerase chain reaction (RT-qPCR) analyses were performed using SYBR GREEN qPCR Super Mix (Invitrogen, Waltham, MA, USA). We used three replicates for the RT-qPCR experiments. Furthermore, we calculated the relative expression level of RNA using the comparative cycle threshold (CT) method (2^–ΔΔCt^) and circRNAs were subjected to GAPDH as an internal control. The primer sequences are shown in Table 1.

**Table 1.** Primers used in RT-qPCR analysis.

Accession No.	Primer sequence (5’-3’)	The size of production (bp)
mmu-circ-0081343-	F: GCTCCCGGGTCATTTCCATC R: GCGTCCACACATAGGCATTG	118bp
GAPDH	F: GGCCTCCAAGGAGTAAGAAA R: GCCCCTCCTGTTATTATGG	141bp

### Western blotting analysis

We collected placental tissues using a RIPA buffer (Invitrogen, Shanghai, China) and calculated the protein concentration using a bicinchoninic acid (BCA) protein assay kit (ab207002, Abcam). Furthermore, proteins were extracted using 10 % sodium dodecyl sulfate-polyacrylamide gels (SDS-PAGE) and transferred to polyvinylidene fluoride (PVDF) membranes. The membranes were blocked with 5 % skim milk and incubated overnight with primary antibodies, including anti-beclin1 (dilution 1:1000, ab128874, Abcam), anti-P62 (dilution 1:1000, ab38898, Abcam), anti-PI3 K (dilution 1:1000, 2207s, CST, USA), and anti-AKT (dilution 1:1000, 2643s, CST, USA) antibodies. Subsequently, the membranes were incubated with secondary antibodies at room temperature. We identified the blots using an enhanced chemiluminescence reagent, and performed quantification of individual protein bands using Image Pro-Plus 6.0.

### Hematoxylin and eosin staining

Placentas were sectioned after embedding in paraffin and stained with hematoxylin and eosin (H&E) following standard procedures. Sections with a thickness of 5 μm were deparaffinized in xylene, rehydrated using a progressive series of ethanol, and then cleaned with water. The sections were rinsed in a weakly acidic solution to remove any remaining stain, after hematoxylin staining. Furthermore, the tissue was counterstained with eosin Y after hematoxylin was applied. We measured the areas of the junctional zone and labyrinth layer of the placenta using the ImageJ software.

### Immunohistochemistry

Paraffin-embedded sections were deparaffinized and microwave-heated in 10 mM sodium citrate buffer (pH 6.0) for 10 min to retrieve the antigen before cooling to room temperature. Endogenous horseradish peroxidase (HRP) was rendered inactive by incubation with hydrogen peroxide for ten minutes. Sections were blocked for 1 h with 10% goat serum buffer (Beyotime, Shanghai, China) to avoid nonspecific binding, and then primary antibodies were incubated at 4 °C for the entire night. The sections were then incubated with secondary antibodies for 1 h at room temperature, washed with phosphate-buffered saline (PBS), stained with 3,3'-diaminobenzidine solution (DAB), and examined under a microscope to capture images.

### Fetal growth restriction mouse model

C57BL/6J mice were kept under a light/dark cycle (12 h light/12 h dark) in an animal room at a controlled temperature of 22–24 °C and 60–70% relative humidity. All animal procedures were conducted following the guidelines for the Use of Laboratory Animals from the National Institutes of Health. Additionally, the animal experiments performed, followed the policies of the Southern Medical University (Guangzhou, China). The female mice were mated with fertile male mice, and an early plug detection indicated that the embryos were on day 0.5 of pregnancy.

In addition, a mouse model of FGR was established under hypoxic conditions (10.5% O_2_) from maternal gestational day (GD) 11–17.5 (Wu et al. 2015). The pregnant mice in the control group were provided with normoxic housing throughout the gestation period. Notably, Mingkong Biotechnology Co., Ltd. (Guangzhou, China) generated the mmu_circ_0081343 overexpression adenovirus (1 × 10^9^ plaque-forming units [PFUs]/ml) and its antisense control. Four groups of mice (six mice each) were randomly assigned to the Control, FGR, FGR + Ad-null, and FGR + Ad-mmu-circ_0081343 groups. Caudal vein injection of Ad-mmu_circ_0081343 or Ad-null was performed at GD5.

### Fetal and placental measurements

We sacrificed all dams and recorded maternal weights on GD18.5. Furthermore, we recorded the numbers of each litter. Live fetuses and placentas were weighed, and the fetal crown-rump length was determined. In addition, the gross anatomy of the pups was evaluated.

### Statistical analysis

Statistical analyses were performed using the Statistical Package for Social Sciences 18.0 software, and GraphPad Prism 6 was used for graphing (GraphPad, La Jolla, CA, USA). Standard deviation (SD) was used to express the data. Differences between groups were evaluated using One-way analysis of variance, followed by Tukey’s post hoc test or a 2-tailed Student’s t-test. We performed a minimum of three replicates for each experiment and statistical significance was set at *p* ≤ 0.05.

## Result

1. Up-regulation of mmu_circ_0081343 in placental tissue of mouse model of fetal growth restriction

**Figure 1.**
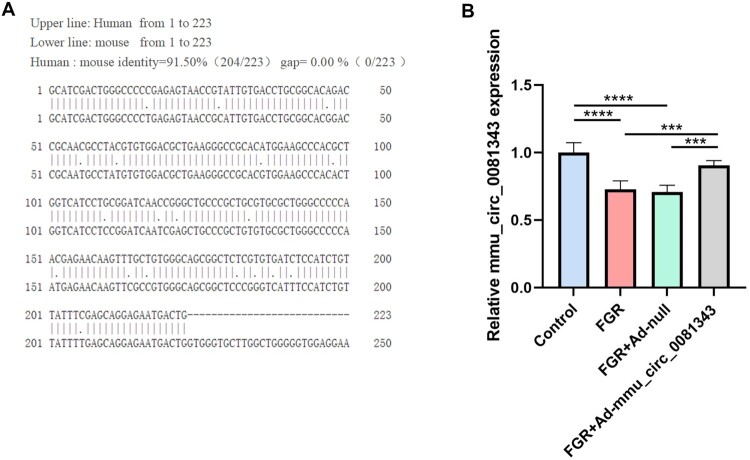
mmu_circ_0081343 was overexpressed in Hypoxia-induced mice model of FGR. (A) Homology of mmu_circ_0081343 in H. sapiens and M. musculus. (B)The expression level of mmu_circ_0081343 in the placenta of fetal growth restriction (FGR) mice was measured using RT-qPCR. Data are presented as mean ± S.E.M., n = 6 per group; * *p* < 0.05, ** *p* < 0.01, *** *p* < 0.001.

To determine the sequence homology of circ_0081343 between humans and rodents, we used the EMBOSS Needle tool for analysis. The results showed no homology between humans and rats, while humans and mice exhibited 91.50% sequence homology for circ_0081343 (Figure 1A). RT-qPCR analysis was conducted to further evaluate the expression of mmu_circ_0081343 in the mice placentas. Consequently, mmu_circ_0081343 expression was reduced in the placentas of the FGR group compared with that in the control group. Furthermore, mmu_circ_0081343 expression significantly increased after mmu_circ_0081343 treatment (Figure 1B).

2. mmu_circ_0081343 improves fetal growth restriction-related phenotypes induced by hypoxia in mice

**Figure 2.**
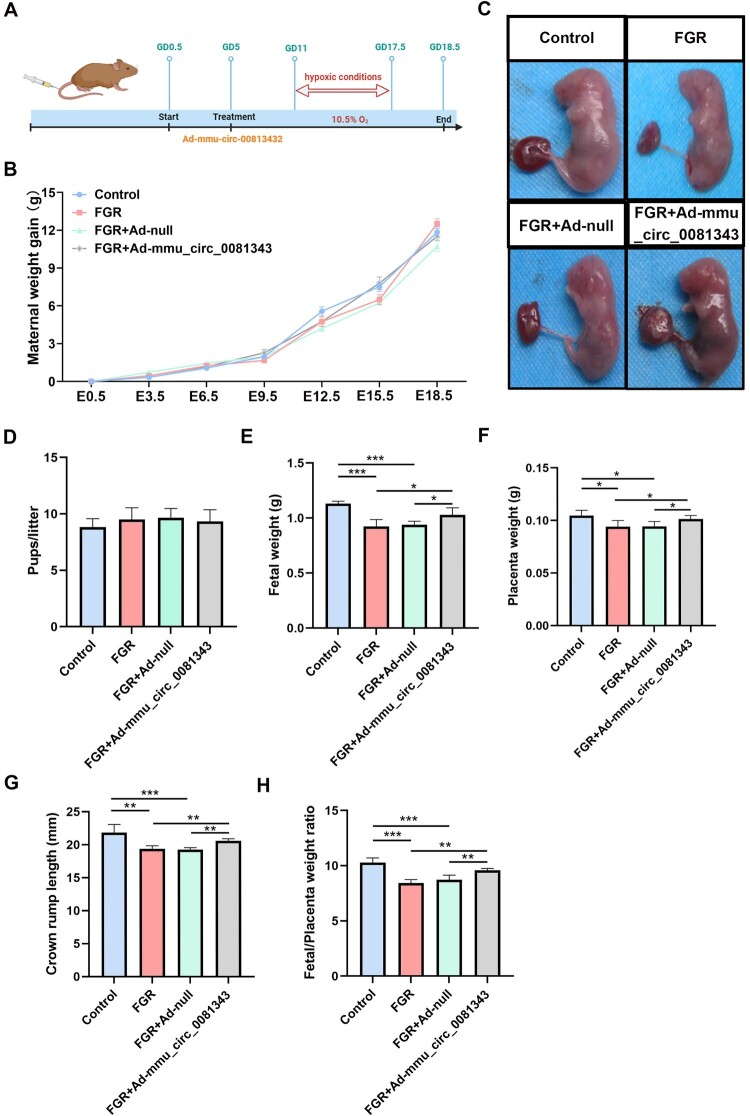
Effect of mmu_circ_0081343 supplementation on fetal and placental growth in each group. (A) Overview of the schematic timeline of the mouse experiment. (B) Maternal weight gain, (C) representative images of fetuses and placentas in each group, (D) pups/litter, (E) average fetal weight on E18.5, (F) average placental weight on E18.5, (G) average crown-rump length on E18.5, and (H) fetal to placental weight ratio on E18.5. Data are presented as mean ± S.E.M., n = 6 per group; * *p* < 0.05, ** *p* < 0.01, *** *p* < 0.001.

To evaluate the impact of circ_0081343 overexpression on pregnancy outcomes, we established a mouse model of FGR under hypoxic condition. The overall timeline of the study with mmu_circ_0081343 supplementation is shown in Figure 2A. The weight gain of pregnant mice was comparable between the groups (Figure 2B). Images of fetuses from each group are shown in Figure 2C. The number of pups per litter did not differ significantly between the groups (Figure 2D). However, we observed a significant reduction in fetal weight, placental weight, and crown-rump length in the FGR and FGR + Ad-null groups compared with the control group (Figure 2E-G). Placental efficiency, commonly defined as the fetal-to-placental weight ratio, is widely used as a retrospective indicator to evaluate fetal development. The FGR mouse model showed a decline in placental efficiency, which was measured as the ratio of pup to placental weight (Figure 2H). Furthermore, the hypoxic status of mouse pups significantly increased after mmu_circ_0081343 injection. These results reveal that the administration of mmu_circ_0081343 to FGR mouse improved placental efficiency and promoted fetal growth.

3. mmu_circ_0081343 supplement improves poor placental pathophysiology in murine placentas with hypoxia-induced fetal growth restriction

**Figure 3.**
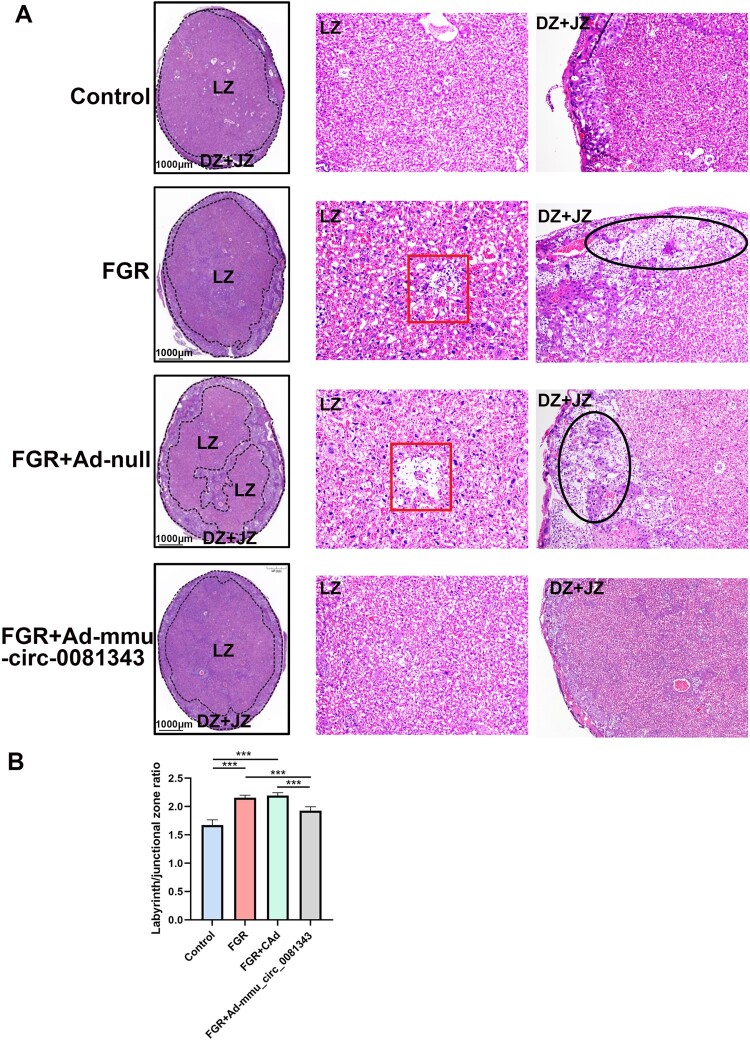
Staining images of mouse placental structure in each group. (A) H&E staining murine placentas from control, fetal growth restriction (FGR), FGR + Ad-null, and FGR + Ad-mmu _circ_0081343 group. (B) Ratio of junction area/labyrinth area in each group at E18.5. Left: General view of the placenta at low magnification (× 20); scale bar = 1 mm. Right: representative image of decidua zone and junction zone at high magnification (× 400); scale bar = 250μm. Red square: perivascular edema. Black oval: villous interstitial edema. Data are presented as mean ± S.E.M., n = 6 per group; * *p* < 0.05, ** *p* < 0.01, *** *p* < 0.001.

To examine placental pathology, we performed H&E staining to assess structural changes in the placentas of each group. The mouse placenta is organized into three structurally and functionally distinct regions, each playing a specialized role in fetal development: the junctional (JZ), labyrinth (LZ), and decidua (DZ). The labyrinth zone, which has rich maternal and fetal blood flow, is the primary site of maternal-fetal exchange. The LZ in the control group was widely dispersed and had abundant blood flow, whereas the DZ, JZ, and LZ structures were all clearly defined. In the FGR and FGR + Ad-null groups, the proportions of DZ and JZ were significantly higher, whereas the proportion of LZ was significantly lower. In addition, most decidual cells showed lysis, necrosis, and hyaline degeneration, resulting in vacuole-like changes. The trophoblast cell size in the LZ also varied, with apoptotic cells dispersed throughout. Interstitial edema was also observed within the vascular zone. Placental pathology in the FGR + mmu_circ_0081343 group was comparable to that in the control group, as shown in Figure 3.

4. mmu_circ_0081343 treatment increases autophagic activity in murine placentas with hypoxia-induced fetal growth restriction

**Figure 4.**
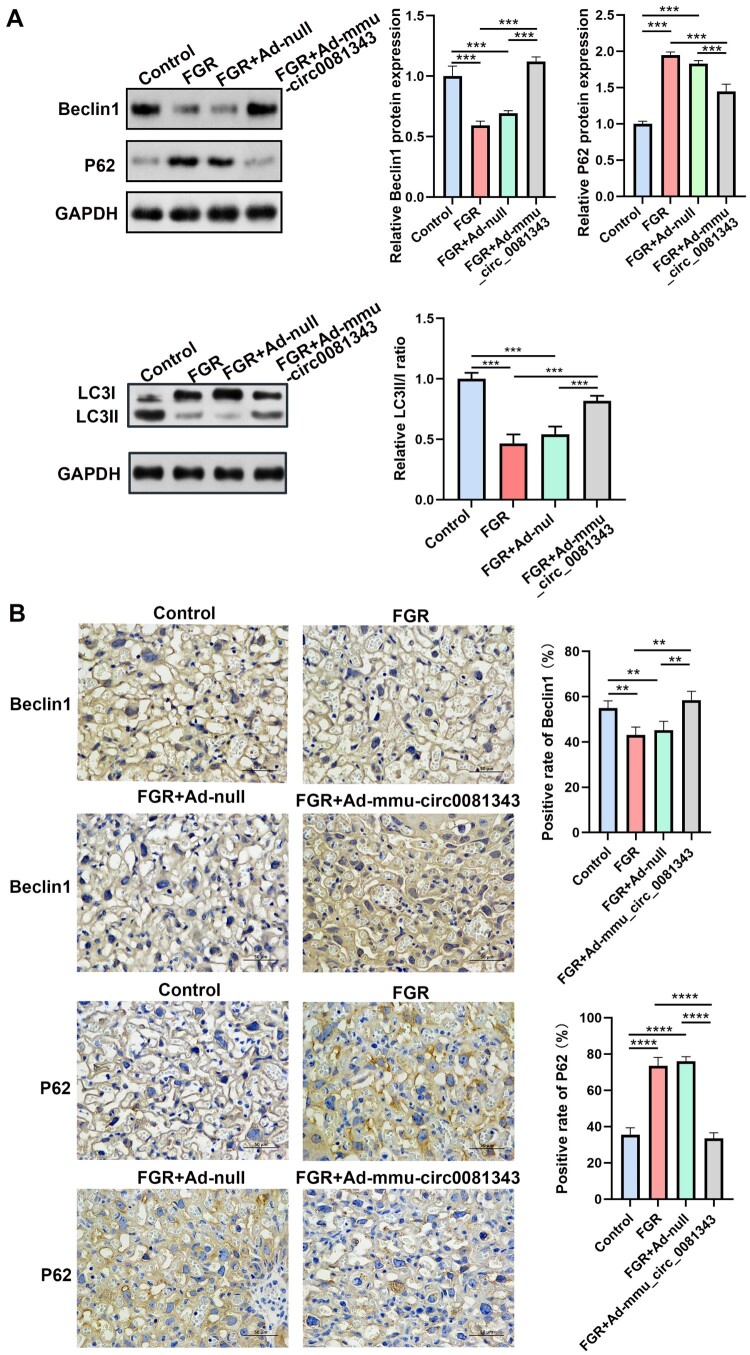
Overexpression of mmu_circ_0081343 promote autophagy in FGR placental tissue. (A) Effect of mmu_circ_0081343 overexpression on the expression of autophagy-related proteins (Beclin1, LC3II/I and SQSTM1/p62) was detected by western blotting in control, FGR, FGR + Ad-null, and FGR + Ad-mmu_circ_0081343 groups. (B) Effect of mmu_circ_0081343 overexpression on the expression of autophagy-related proteins (Beclin1, LC3II/I and SQSTM1/p62) was detected by immunohistochemistry in control, FGR, FGR + Ad-null, and FGR + Ad-mmu_circ_0081343 groups. Data are presented as mean ± S.E.M., n = 6 per group; * *p* < 0.05, ** *p* < 0.01, *** *p* < 0.001.

To determine autophagic activity in the placenta, we examined autophagy-related proteins in placental tissues using western blotting analysis. The results showed that Beclin1 protein expression was significantly reduced in the FGR and FGR + Ad-null groups compared to the control group, while SQSTM1/p62 protein expression was markedly increased. Notably, after treatment with Ad-mmu_circ_0081343, Beclin1 protein levels were elevated, and SQSTM1/p62 protein levels were significantly reduced compared to the FGR and FGR + Ad-null groups (Figure 4A). The immunohistochemical results were consistent with those of the western blotting, as shown in Figure 4B. Collectively, these data demonstrate that mmu_circ_0081343 overexpression effectively enhances autophagy in the FGR model.

5. mmu_circ_0081343 administration inhibited pyroptosis in placentas with hypoxia-induced fetal growth restriction mouse model

**Figure 5.**
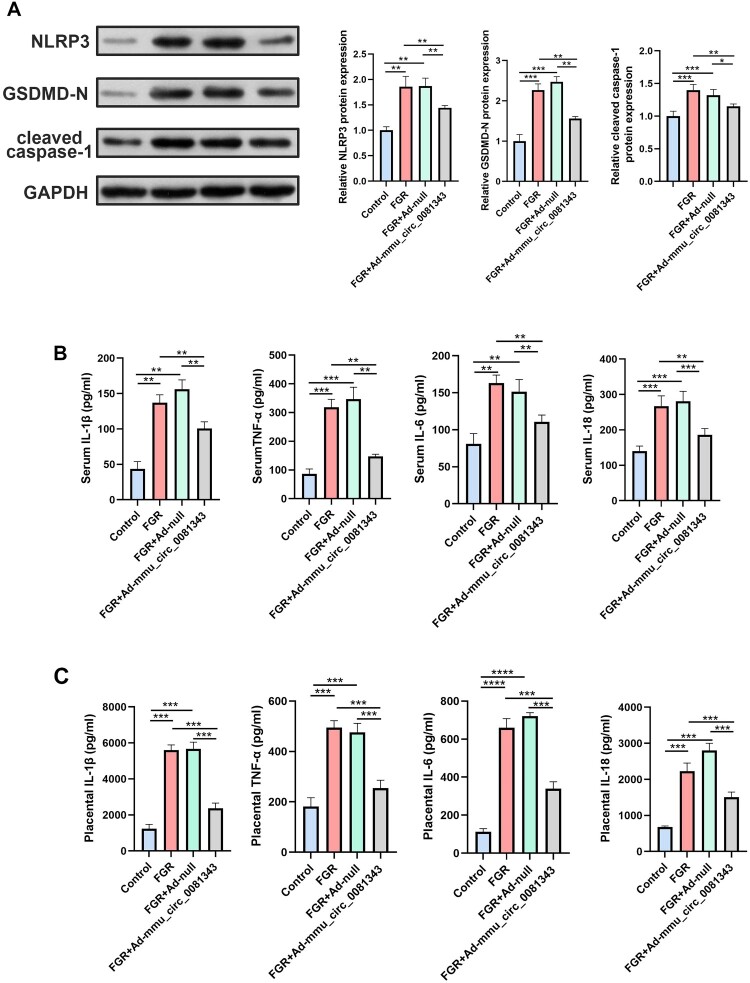
Overexpression of mmu_circ_0081343 attenuated pyroptosis in Fetal growth restriction placental tissue. (A) The effect of mmu_circ_0081343 overexpression on the expression of pyroptosis-related proteins (NLRP3, cleaved caspase-1, and GSDMD-N) was detected by western blotting in the control, FGR, FGR + Ad-null, and FGR + Ad-mmu_circ_0081343 groups. Effect of serum (B) and placental (C) mmu_circ_0081343 overexpression on the expression of inflammatory factors, including interleukin (IL)−1β, IL-6, IL-18 and tissue necrotic factor-α (TNF-α) was detected by enzyme-linked immunosorbent assay assays in control, fetal growth restriction (FGR), FGR + Ad-null, and FGR + Ad-mmu_circ_0081343 groups. Data are presented as mean ± S.E.M., n = 6 per group; * *p* < 0.05, ** *p* < 0.01, *** *p* < 0.001.

To assess the effect of mmu_circ_0081343 on pyroptosis in hypoxia-induced FGR, we analyzed the expression levels of pyroptosis-related proteins (NLRP3, cleaved caspase-1, and GSDMD-N) by western blotting and measured inflammatory cytokines (TNF-α, IL-1β, IL-6, and IL-18) by ELISA in each group. The results revealed that the expression levels of NLRP3 and cleaved caspase-1 were significantly elevated in hypoxia-induced mouse placentas, but this increase was attenuated by mmu_circ_0081343 injection (Figure 5A). Moreover, the mmu_circ_0081343 treatment group exhibited a significant reduction in GSDMD-N expression, a key executor of pyroptosis, compared to the FGR and FGR + Ad-null groups. Additionally, mmu_circ_0081343 upregulation suppressed the levels of pro-inflammatory cytokines (TNF-α, IL-1β, IL-6, and IL-18) in both hypoxia-induced mouse serum (Figure 5B) and placental tissues (Figure 5C). These findings suggest that mmu_circ_0081343 treatment significantly inhibits NLRP3 inflammasome-mediated pyroptosis in the FGR mode.

6. mmu_circ_0081343 overexpression upregulates autophagy and reduces pyroptosis by inactivating the PI3 K/AKT/HIF-1α pathway

**Figure 6.**
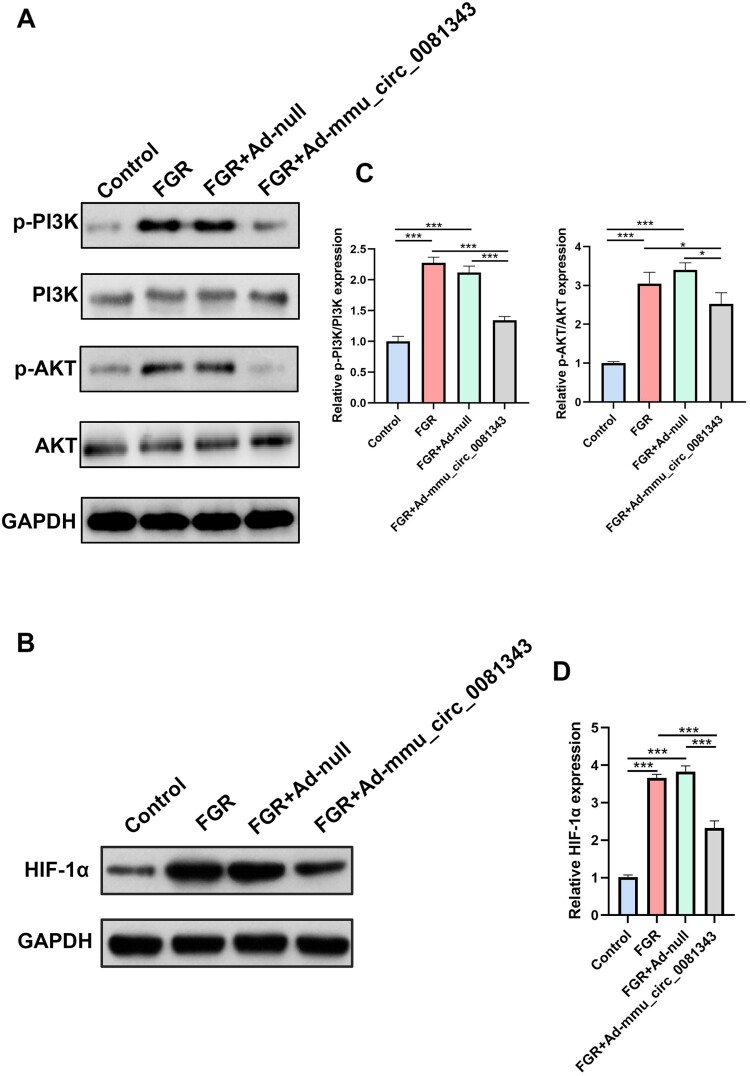
mmu_circ_0081343 inactivates the PI3 K/AKT/HIF-1α signaling pathway. (A and B) Western blotting for the expression of constituents of the phosphoinositide 3-kinase (PI3 K)/AKT signaling pathway and its corresponding downstream hypoxia-inducible factor (HIF)−1α with overexpression of mmu_circ_0081343 in control, fetal growth restriction (FGR), FGR + Ad-null, and FGR + Ad-mmu_circ_0081343 groups. The gels were run under the same experimental conditions and cropped blots are shown. (C and D) The optical density values of the the PI3 K, p-PI3 K, AKT, p-AKT, and HIF-1α expression levels, normalized to the loading control glyceraldehyde 3-phosphate dehydrogenase. Data are presented as mean ± S.E.M., n = 6 per group; * *p* < 0.05, ** *p* < 0.01, *** *p* < 0.001.

To explore the molecular mechanisms underlying the effects of mmu_circ_0081343 on autophagy and pyroptosis, we analyzed the activity of the PI3 K/AKT/HIF-1α pathway following hypoxia exposure. Western blotting analysis revealed that the phosphorylation levels of PI3 K and AKT (Figure 6A and C) and the expression of HIF-1α (Figure 6B and D) were significantly reduced in placental tissues of the FGR and FGR + Ad-null groups compared to the control group. However, after Ad-mmu_circ_0081343 administration, the phosphorylation levels of PI3 K and AKT, as well as HIF-1α expression, were significantly increased compared to the FGR and FGR + Ad-null groups. Notably, the total protein levels of PI3 K and AKT remained unchanged across all experimental groups (Figure 6A and C). These results suggest that mmu_circ_0081343 improves pregnancy outcomes and alleviates placental injury in the FGR mouse model by modulating the PI3 K/AKT/HIF-1α pathway, which regulates autophagy and pyroptosis.

## Discussion

Currently, only a limited number of effective pharmacological agents have been developed for the treatment of FGR and pregnancy termination or early delivery of the fetus and placenta remains the most definitive treatment option. Consequently, recent research has focused on identifying regulatory molecules, such as circRNAs, which have been shown to play critical roles in the development of FGR and its underlying molecular mechanisms. Wang et al. identified 244 differentially expressed circRNAs in FGR placentas, of which 100 were upregulated and 144 were downregulated. Among these, circ_0081343, one of the top six differentially expressed circRNAs, was significantly downregulated in human placenta (Wang et al. 2020). Mechanistic studies revealed that circ_0081343 promotes trophoblast cell migration and invasion while suppressing apoptosis by sponging miR-210-5p (Wang et al. 2021). Furthermore, circ_0081343 regulates trophoblast cell autophagy by mediating the nuclear translocation of Rbm8a (Zheng et al. 2024). These findings suggest that circ_0081343 may contribute to the etiology and pathogenesis of FGR. Therefore, we further investigated the functional role of circ_0081343 in mice to explore its potential as a biomarker or therapeutic target using in vivo animal models of FGR.

Previous studies in pregnant rodents have demonstrated that late-onset hypoxia during the third trimester of gestation significantly induces fetal growth restriction (FGR) (Jang et al. 2015; Francis et al. 2020). Based on these findings, we established a mouse model of FGR by exposing pregnant mice to hypoxia (10.5% O_2_) from gestational day (GD) 11 to GD 17.5. Notably, circ-0081343 shows high genetic homology between humans and mice, and its expression was significantly downregulated in the placental tissues of FGR mice. These results suggest that insights gained from the mouse model are likely translatable to humans.

In this study, maternal hypoxia successfully induced FGR, as demonstrated by significant reductions in fetal weight, placental weight, and the fetal-to-placental weight ratio, consistent with previous reports (Thompson et al. 2016; Weng et al. 2022). These results effectively recapitulate the placental developmental abnormalities characteristic of FGR, confirming the reliability of our model. Additionally, FGR placentas exhibited a smaller labyrinth zone, an expanded junctional zone, and pathological lesions compared to controls. Strikingly, supplementation with mmu-circ_0081343 not only increased fetal weight, placental weight, and the fetal-to-placental weight ratio but also restored labyrinth zone thickness and alleviated pathological damage. These findings demonstrate that mmu-circ_0081343 overexpression significantly enhances placental function and improves pregnancy outcomes in hypoxic FGR mice.

Autophagy is critically involved in embryogenesis, implantation, and the maintenance of pregnancy (Nakashima et al. 2019). Nevertheless, the role of autophagy in human and mouse placentas affected by FGR remains controversial. On one hand, increased autophagic vacuoles have been observed in the syncytiotrophoblast layer of human FGR placentas compared to normal placentas, suggesting autophagy activation (Curtis et al. 2013). Consistent with this, elevated expression of autophagy-related proteins, including LC3B-II and beclin-1, has been reported in FGR placentas (Hung et al. 2012). On the other hand, studies in mice have demonstrated that knockout of autophagy-related genes impairs fetal growth, indicating a potential association between FGR and autophagy inhibition (Kojima et al. 2015). For example, Muralimanoharan et al. found that Atg7-/- mice, which lack Atg7 – a key regulator of autophagosome biogenesis-exhibited significantly reduced offspring birth weight (Muralimanoharan et al. 2016). Beclin-1, another critical autophagy regulator, plays a pivotal role in autophagy initiation and autophagosome formation (Liang et al. 1999). Additionally, the degradation of P62, a well-established autophagic substrate, further underscores the dynamic regulation of autophagic processes (Lee and Weihl 2017). The ratio of LC3-II/I can estimate autophagy levels. Our previous study demonstrates that circ_0081343 overexpression in vitro significantly enhanced autophagic flux, characterized by elevated LC3-II/LC3-I ratios, decreased p62 levels, and increased autophagosome counts (Zheng et al. 2024). The results of our study revealed a significant decrease in the LC3-II/I ratio and beclin-1 expression, accompanied by elevated levels of P62, in placental tissues under hypoxic conditions. However, treatment with mmu_circ_0081343 effectively reversed these changes, aligning with our in vitro findings (Zheng et al. 2024). These results demonstrate that mmu_circ_0081343 significantly alleviates autophagy dysfunction in the FGR mouse model.

The NLRP3 inflammasome, a multiprotein complex comprising NLRP3, procaspase-1, and apoptosis-associated speck-like protein (ASC), induces pyroptosis by activating gasdermin D-N (GSDMD-N), a pore-forming protein that disrupts the plasma membrane and drives programmed cell death (Mangan et al. 2018). A previous study reported that the inflammasome activation at the maternal-fetal interface participates in pyroptosis and that inflammatory reactions during FGR are regulated by the NLRP3 inflammasome (Alfian et al. 2022). Therefore, pregnancy-related diseases that involve the inactivation of this inflammasome process can be managed by downregulating the expression of key components of the NLRP3 inflammasome or pharmacological inhibition of the inflammasome (Li et al. 2021). Our study showed that mmu-circ_0081343 administration significantly inhibits placental pyroptosis in FGR mouse by downregulating key NLRP3 inflammasome components (NLRP3 and cleaved caspase-1), suppressing GSDMD-N expression, and attenuating the release of pro-inflammatory cytokines, including IL-1β, IL-18, IL-6, and TNF-α.

The PI3 K/AKT signal pathway plays an important role in regulating cell growth and survival, has been described to be activated under hypoxia in the placenta (Feng et al. 2022). As a central regulator of hypoxic signaling, HIF-1α overexpression in the placenta disrupts tissue architecture, impairs lineage specification, and suppresses trophoblast differentiation (Albers et al. 2019). It has reported HIF-1α is subjected to regulation by the PI3 K/AKT pathway. Specifically, the stability of HIF-1α is closely associated with PI3 K/AKT activity (Chen et al. 2001), and its expression can be significantly reduced by PI3 K inhibitors (Jiang et al. 2001). These data suggested that hypoxia can not only activate the PI3 K/AKT signaling pathway but can also promote the expression of HIF-1α. Moreover, previous studies have indicated that the PI3 K/AKT/HIF-1α pathway serves as the major regulator of autophagy (Duan et al. 2020) and pyroptosis (Gao et al. 2023). In our study, FGR mouse exhibited a significant suppression of the PI3 K/AKT signaling pathway and a marked reduction in HIF-1α protein expression. However, treatment with mmu-circ_0081343 partially restored PI3 K/AKT signaling activity and increased HIF-1α protein expression, suggesting its potential role in mitigating the pathological mechanisms underlying FGR.

In conclusion, our findings demonstrate that circ_0081343 significantly improves pregnancy outcomes and alleviates pathological changes in a mouse model of hypoxia-induced FGR. Mechanistically, based on previous in vitro findings, circ_0081343 exerts its protective effects by promoting autophagy and inhibiting pyroptosis, likely through modulation of the PI3 K/AKT/HIF-1α signaling pathway. Therefore, our study provides that mmu-circ_0081343 may serve as a promising therapeutic target for mitigating the progression of FGR in vivo.

## Ethics approval and consent to participate

The study was approved by Southern Medical University (Guangzhou, China) and all animals were treated according to the Use of Laboratory Animals of the National Institutes of Health.

## Data Availability

Data will be made available on request.

## Abbreviations

**Abbreviations:** AKT: serine/threonine protein kinase B; circRNAs: circular RNAs; FGR: fetal growth restriction; GSDMD-N: N-terminal fragment of membrane pore-forming gasdermin; HIF-1α: hypoxia inducible factor-1α; IL-6: interleukin-6; IL-18: interleukin 18; IL-1β: interleukin-1β; NLRP3: NOD-like receptor protein 3; PI3K: phosphatidylinositol 3-kinase; TNF-α: Tumour Necrosis Factor alpha
